# Exploring untapped bacterial communities and potential polypropylene-degrading enzymes from mangrove sediment through metagenomics analysis

**DOI:** 10.3389/fmicb.2024.1347119

**Published:** 2024-04-04

**Authors:** Onnipa Pawano, Nuttarin Jenpuntarat, Wolfgang R. Streit, Pablo Pérez-García, Thunyarat Pongtharangkul, Pranee Phinyocheep, Parinda Thayanukul, Jirayut Euanorasetr, Bungonsiri Intra

**Affiliations:** ^1^Department of Biotechnology, Faculty of Science, Mahidol University, Bangkok, Thailand; ^2^Mahidol University and Osaka Collaborative Research Center on Bioscience and Biotechnology, Bangkok, Thailand; ^3^Department of Microbiology and Biotechnology, University of Hamburg, Hamburg, Germany; ^4^Molecular Microbiology, Institute of General Microbiology, Kiel University, Kiel, Germany; ^5^Department of Chemistry, Faculty of Science, Mahidol University, Bangkok, Thailand; ^6^Department of Biology, Faculty of Science, Mahidol University, Bangkok, Thailand; ^7^Faculty of Science, Center of Excellence for Vectors and Vector-Borne Diseases, Mahidol University at Salaya, Nakhon Pathom, Thailand; ^8^Laboratory of Biotechnological Research for Energy and Bioactive Compound (BREBC), Department of Microbiology, Faculty of Science, King Mongkut's University of Technology Thonburi, Bangkok, Thailand

**Keywords:** plastic biodegradation, microbial community, metagenomics analysis, polypropylene, mangrove sediment

## Abstract

The versatility of plastic has resulted in huge amounts being consumed annually. Mismanagement of post-consumption plastic material has led to plastic waste pollution. Biodegradation of plastic by microorganisms has emerged as a potential solution to this problem. Therefore, this study aimed to investigate the microbial communities involved in the biodegradation of polypropylene (PP). Mangrove soil was enriched with virgin PP sheets or chemically pretreated PP comparing between 2 and 4 months enrichment to promote the growth of bacteria involved in PP biodegradation. The diversity of the resulting microbial communities was accessed through 16S metagenomic sequencing. The results indicated that Xanthomonadaceae, unclassified Gaiellales, and Nocardioidaceae were promoted during the enrichment. Additionally, shotgun metagenomics was used to investigate enzymes involved in plastic biodegradation. The results revealed the presence of various putative plastic-degrading enzymes in the mangrove soil, including alcohol dehydrogenase, aldehyde dehydrogenase, and alkane hydroxylase. The degradation of PP plastic was determined using Attenuated Total Reflectance Fourier Transform Infrared Spectroscopy (ATR-FTIR), Scanning Electron Microscopy (SEM), and Water Contact Angle measurements. The FTIR spectra showed a reduced peak intensity of enriched and pretreated PP compared to the control. SEM images revealed the presence of bacterial biofilms as well as cracks on the PP surface. Corresponding to the FTIR and SEM analysis, the water contact angle measurement indicated a decrease in the hydrophobicity of PP and pretreated PP surface during the enrichment.

## GRAPHICAL ABSTRACT

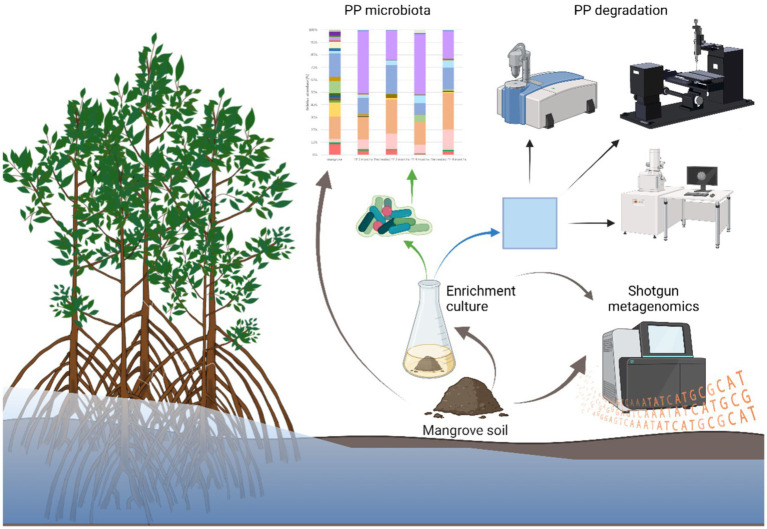

## Highlights

- Enrichment culture of mangrove soil with PP polymer promoted the growth of PP-degrading bacteria.
- The bacterial community composition was altered after enrichment for two and 4 months.
- Diverse bacteria in mangrove sediments showed PP-degrading potential based on FTIR, water contact angle, and SEM results.
- The dominant bacterial families were *Xanthomonadaceae*, one unclassified family within *Gaiellales*, *Nocardioidaceae*, one unclassified family within *Solirubrobacterales*, and *Pseudomonadaceae*.
- Shotgun metagenomics revealed enrichment of potential PP-degrading enzymes, including alcohol dehydrogenase, aldehyde dehydrogenase, and alkane hydroxylase.

## 1 Introduction

Plastic is an economically important material with a wide range of applications (Shah et al., 2008). The global production of plastic has been steadily increasing each year, reaching 390 million tons in 2021 (Plastic Europe, 2020). The ubiquity of plastic use is due to characteristics such as light weight, stability and durability, transparency, ease of modification, and low production cost (Akan et al., 2021; Ali et al., 2021). Polypropylene (PP) is the second-most produced plastic, accounting for 19.3% of global production (Plastic Europe, 2020). PP is highly utilized in packaging materials that are considered single-use plastics, and it is anticipated that over 80% of these materials will eventually end up as waste (Mohanan et al., 2020; Lee et al., 2023). Since PP consists of a linear chain of propylene monomers, it exhibits strong resistance to environmental degradation, giving rise to significant environmental concerns that impact ecosystems (Urbanek et al., 2017). Microorganisms can accommodate nearly every type of environment, and they can use a wide variety of compounds as carbon sources, even complex advanced polymers such as petroleum-derived plastics (Purohit et al., 2020; Yuan et al., 2020). In recent years, research studies have focused attention on the biodegradation of plastic, since this is a potentially eco-friendly and affordable method (Taghavi et al., 2021). Biodegradation of plastic is a complex process comprising four main steps: microbial colonization and biofilm formation, biodeterioration by microbial enzymes, biofragmentation of polymers into lower-weight polymers, and mineralization by the intracellular pathways of microorganisms (Auta et al., 2017; Ganesh Kumar et al., 2020). Several studies have investigated the microorganisms and enzymes involved in plastic biodegradation. For example, *Ideonella sakaiensis* 201-F6, which was isolated from a PET debris-contaminated environment, possesses PETase capable of nearly completely degrading PET film within 6 weeks (Yoshida et al., 2016). The hydrocarbon-degrading bacterium *Alcanivorax borkumensis* was able to degrade LDPE, with a percentage of weight loss at 3.4% after 80 days (Delacuvellerie et al., 2019). Plastic-degrading strains of *Bacillus cereus* and *Bacillus gottheilii* isolated from mangrove ecosystems have shown the potential to degrade PE, PET, PP, and PS after 40 days of incubation (Auta et al., 2017). However, biodegradation of the most widely used polymers such as PP has not been as well researched, with only a few reports available concerning microbial-mediated PP degradation (Andler et al., 2022).

Mangrove ecosystems host a rich diversity of microorganisms due to their location in intertidal zones along tropical and sub-tropical coastlines. These zones offer favorable environmental conditions for microbial growth, including high temperatures, elevated salinity, pH levels, and organic matter content (Auta et al., 2017; Liu et al., 2023). Pseudomonadota (née Proteobacteria), Bacteroidota (née Bacteroidetes), Chloroflexota (née Chloroflexi), Actinomycetota (née Actinobacteria), Parvarchaeota, Acidobacteriota (née Acidobacteria) and Cyanobacteria were observed as the principle bacterial phyla in the mangrove sediments from Guangxi province, China (Gong et al., 2019). However, mangrove ecosystems also serve as effective traps for marine plastic debris. The plants capture and entangle plastic waste originating from nearby land and ocean areas due to the presence of well-developed pneumatophores (aerial roots) (Cordova et al., 2021; Deng et al., 2023). Currently, mangroves face threats from human activities, including plastic pollution (Liu et al., 2023). Plastic waste creates a distinct niche for microbes in various environments (Delacuvellerie et al., 2019; Yu et al., 2023). Since 2013, term “plastisphere” was coined as microbial diversity resided on the plastic marine debris (Zettler et al., 2013). Exploration of microbial-mediated plastic degradation in different environments or through enrichment cultures to investigate dynamic changes of microbial communities is the common strategy (Sun et al., 2023; Li et al., 2024). However, there is a limited number of studies specifically focusing on polypropylene (PP).

Therefore, the objective of this study was to investigate the changes in the bacterial community when mangrove sediment was enriched with either PP or chemically pretreated PP. The pretreated PP could be more susceptible to microbial degradation than virgin PP due to the presence of polar functional groups resulting from the chemical pretreatment process.

Additionally, the research aimed to explore the microbial enzymes involved in plastic degradation within the mangrove sediment. The composition of the bacterial community colonizing on the plastic surface was accessed by 16S metagenomic sequencing, while plastic-degrading enzymes were investigated using mangrove solid sediment through shotgun metagenomics. The degradation of the PP and pretreated PP polymers was analyzed by Fourier transform infrared spectroscopy (FTIR) and Field Emission Scanning Electron Microscopy (FE-SEM). Understanding the diversity of bacteria associated with PP can offer valuable insights for assessing the biodegradation of PP plastics within the marine environment.

## 2 Materials and methods

### 2.1 Sediment collection and plastic preparation

Mangrove sediment was collected from under a mangrove tree in Chanthaburi, Thailand (12°31.8415′N; 102°3.0207′E) and was stored at 4°C in a refrigerator.

The moplen HP500N polypropylene [cas number: 9003-07-0 (homopolymer)] (HMC Polymers, Thailand) was used and added to Mineral Salts Medium (MSM). PP sheets were prepared by cutting into 2 cm^2^ squares and separating into two types: virgin PP and pretreated PP. Chemical treatment was used to prepare the pretreated PP by immersing the sheets in a solution of 0.25/0.5 mol/L of KMnO_4_/HCl at 45°C for 8 h (Balasubramanian et al., 2014). After that, the PP sheets were rinsed with HCl and distilled water to remove any remaining chemical residues. Before use, both the virgin PP and pretreated PP sheets were sterilized in 70% ethanol overnight and then air dried under sterile conditions.

### 2.2 PP enrichment culture and biofilm collection

The enrichment culture method was adapted from Delacuvellerie et al. (2019). One gram of mangrove wet solid sediment was mixed with 10 mL of sterile synthetic seawater (Thermo Scientific, United States), shaken for 2 min, and then allowed to settle. After that, 300 μL of the supernatant was transferred to 15 mL of MSM medium (2.27% K_2_HPO_4_, 0.095% KH_2_PO_4_, 0.06% NH_4_SO_4_, and 0.2% metal solution [0.637% Na_2_EDTA.2H_2_O, 0.1% ZnSO_4_.7H_2_O, 0.05% CaCl_2_.2H_2_O, 0.25% FeSO_4_.7H_2_O, 0.01% NaMoO_4_.2H_2_O, 0.01% CuSO_4_.5H_2_O, 0.02% CoCl_2_.6H_2_O, 0.052% MnSO_4_.H_2_O, and 6% MgSO_4_.7H_2_O, pH 6.5)]. PP supplemented the carbon sources provided by the mangrove soil. Control samples were PP and pretreated PP in MSM medium without sediment. The cultures were incubated at 30°C and 140 rpm for 2 and 4 months.

After incubation, the plastic samples were collected from the culture media. The biofilms on PP and pretreated PP sheet surfaces were removed by scraping the sheets with the distal end of a sterile spatula and transferred to DNA/RNA Shield™ reagent (DNA/RNA Shield™, Zymo Research, Irvine, CA) for DNA preservation prior to extraction.

### 2.3 DNA extraction

The DNA from microbial biofilms, obtained from enrichment cultures of virgin and pretreated PP, underwent 16S rRNA gene amplicon sequencing. DNA from mangrove solid sediment was used as a control. DNA extractions were carried out using a ZymoBIOMICS^®^-96 MagBead DNA Kit (Zymo Research, Irvine, CA) following the manufacturer’s instructions.

### 2.4 16S rRNA gene amplicon sequencing and data processing

A library of the bacterial 16S ribosomal RNA gene was prepared using the Quick-16STM NGS Library Prep Kit and the Quick-16STM Primer Set (Zymo Research, Irvine, CA) targeting the V3-V4 region of 16S rRNA through real-time PCR. The final PCR products were quantified by using qPCR fluorescence readings and then pooled based on equal molarity. The final pooled library was purified with the Select-a-Size DNA Clean & Concentrator™ (Zymo Research, Irvine, CA), and subsequently quantified with TapeStation^®^ (Agilent Technologies, Santa Clara, CA) and Qubit^®^ (Thermo Fisher Scientific, Waltham, WA). The final library was sequenced using Illumina^®^ MiSeq™ with a v3 reagent kit (600 cycles). The sequencing was performed with a 10% PhiX spike-in.

Unique amplicon sequence variants were inferred from the raw reads using the DADA2 pipeline (Callahan et al., 2016). Potential sequencing errors and chimeric sequences were also removed with the DADA2 pipeline. Taxonomic assignment was performed using Uclust from Qiime v.1.9.1 with the Zymo Research Database, a 16S database that is internally designed and curated, as a reference. Composition visualization and calculation of alpha diversity were performed with Qiime v.1.9.1.

Quantitative real-time PCR was set up with a standard curve. The standard curve was created using 10-fold serial dilutions of plasmid DNA containing one copy of the 16S gene. The primers used were the same as those used in Targeted Library Preparation. The number of gene copies in the reaction for each sample was calculated using the equation generated by the plasmid DNA standard curve. The PCR input volume was used to calculate the number of gene copies per microliter in each DNA sample. The number of genome copies per microliter of DNA sample (genome copies) was calculated by dividing the gene copy number by an assumed number of gene copies per genome (Zymo Research, Irvine, CA). The value used for 16S copies per genome was 4. The amount of DNA per microliter DNA sample (DNA ng) was calculated using an assumed genome size of 4.64 × 10^6^ bp, the genome size of *Escherichia coli*, for 16S samples. The calculation is shown below:

Calculated Total DNA = Calculated Total Genome Copies × Assumed Genome Size (4.64 × 10^6^ bp) × Average Molecular Weight of a DNA bp (660 g/mole/bp) ÷ Avogadros Number (6.022 × 10^23^/mole).

### 2.5 Shotgun metagenomics sequencing and data processing

Mangrove sediment samples underwent Shotgun metagenomics sequencing using the ZymoBIOMICS^®^ Service (Zymo Research, Irvine, CA) for comprehensive metagenomics analysis. Shotgun metagenomics sequencing libraries were prepared using the Nextera^®^ DNA Flex Library Prep Kit (Illumina, San Diego, CA) with up to 100 ng of DNA as input following the manufacturer’s protocol. Internal dual-index 8 bp barcodes with Nextera^®^ adapters (Illumina, San Diego, CA) were utilized. All libraries were quantified using TapeStation^®^ (Agilent Technologies, Santa Clara, CA) and then pooled in equal amounts. The final pooled library was quantified using qPCR, and sequencing was conducted on the Illumina NovaSeq^®^ platform (Illumina, San Diego, CA).

The raw sequence reads obtained from the bioinformatic analyses underwent additional analysis by BGI Co., Ltd. Initially, the quality of the raw reads was assessed using FastQC 0.11.9, and any adapter sequences were subsequently removed using Fastp 0.23.2. The resulting reads were then subjected to *de novo* assembly using MEGAHIT v.1.2.9. Following the assembly, the gene functions of the assembled contigs were predicted using Prokka v.1.14.6. Plastic-degrading enzymes were selected according to Mohanan et al. (2020) and Ru et al. (2020) and the plastics-active enzymes (PAZy) database.^1^ The specific proteins or genes encoding plastic-degrading enzymes were filtered and mapped back to the contig assembly using Bowtie2 2.5.0. The mapped reads were subsequently counted using Samtools 1.17.

### 2.6 Analysis of PP using ATR-FTIR spectroscopy following bacterial degradation

The changes in the chemical structures of the plastics were determined by Attenuated Total Reflectance- Fourier Transform Infrared (ATR-FTIR) spectroscopy (PerkinElmer Frontier, United States) in the frequency range of 4,000–380 cm^−1^. This analysis was performed on all plastic samples treated with microbes as well as the uninoculated control. The bacterial biofilm was removed from the plastic samples by gently scraping with a spatula. Plastic samples were subsequently cleaned by soaking in 70% alcohol overnight and then dry. The FTIR spectra were processed and visualized using Spectragryph v1.2.15 (F. Menges “Spectragryph - optical spectroscopy software,” Version 1.2.15, 2022).^2^

### 2.7 Analysis of alteration in the hydrophobicity of the PP surface

The water contact angle (WCA), an indicator of the hydrophobicity of a plastic surface, was measured by placing a 1 μL droplet of distilled water onto the clean plastic sheet after removing the biofilm. The WCA was measured at three different locations of each plastic sample using a contact angle measurement device (Dropmaster DM-CE1, Kyowa Interface Science, Japan), and WCA images were taken using the same device. The angle between the water droplet and the plastic surface was calculated using FAMAS software. Statistical analysis of WCA data were performed using ANOVA from SPSS software 18.0. Data were visualized using GraphPad Prism 10.0.0.

### 2.8 Determination of the plastic surface by scanning electron microscopy

The surface texture of the plastic samples without removing the bacterial attachment was determined using a Field emission scanning electron microscope (FE-SEM) (Hitachi SU8010, Japan). The incubated and unincubated plastic samples were prepared by a series of ethanol-soaking steps. The samples were soaked in 30% ethanol for 15 min; this was repeated twice, followed by soaking in 35% ethanol for 15 min, also repeated twice. The process was then repeated with gradually increasing ethanol concentrations of 40, 45, 50, 55, 60, 65, 70, 80, 90, and 95%. Afterward, the plastic sheets were transferred to absolute ethanol and dried using a critical point dryer machine (Hitachi, Japan). The dried samples were then mounted onto SEM Sample Stubs using carbon tape and coated with platinum and palladium. Finally, the samples were analyzed under a high-resolution scanning electron microscope to observe their surface characteristics.

## 3 Results and discussion

### 3.1 Bacterial diversity in mangrove sediment and the enrichment culture

The 16S metagenomics sequencing of mangrove sediment and biofilms from the enrichment cultures with PP and pretreated PP yielded a total of 505,602 raw reads. Specifically, there were 103,644 reads from the mangrove sediment, 88,280 reads from the 2-month enrichment culture of PP, and 105,810 reads from the 2-month enrichment culture of pretreated PP. For the 4-month enrichment culture, the numbers of raw reads obtained were 102,094 for PP and 105,774 for pretreated PP. The data underwent processing to remove unwanted and inefficient sequences, resulting in a final set of unique sequences. There were 975 unique sequences from the mangrove sediment. In addition, the numbers of unique sequences from the 2-month and 4-month enrichment cultures of PP and pretreated PP were 115, 113, 104, and 135, respectively (Table 1).

**Table 1:** Read processing summary of PP and pretreated PP enrichment cultures.

Sample	Raw sequences	Trimmed sequences	Sequences after filtering	Final unique sequences
Mangrove soil	103,644	99,240	36,315	975
PP enrichment for 2 months	88,280	84,704	36,735	115
Pretreated PP enrichment for 2 months	105,810	101,978	46,267	113
PP enrichment for 4 months	102,094	97,620	44,008	104
Pretreated PP enrichment for 4 months	105,774	101,946	46,910	135
Total	505,602	485,488	210,235	1,442

**Figure 1:**
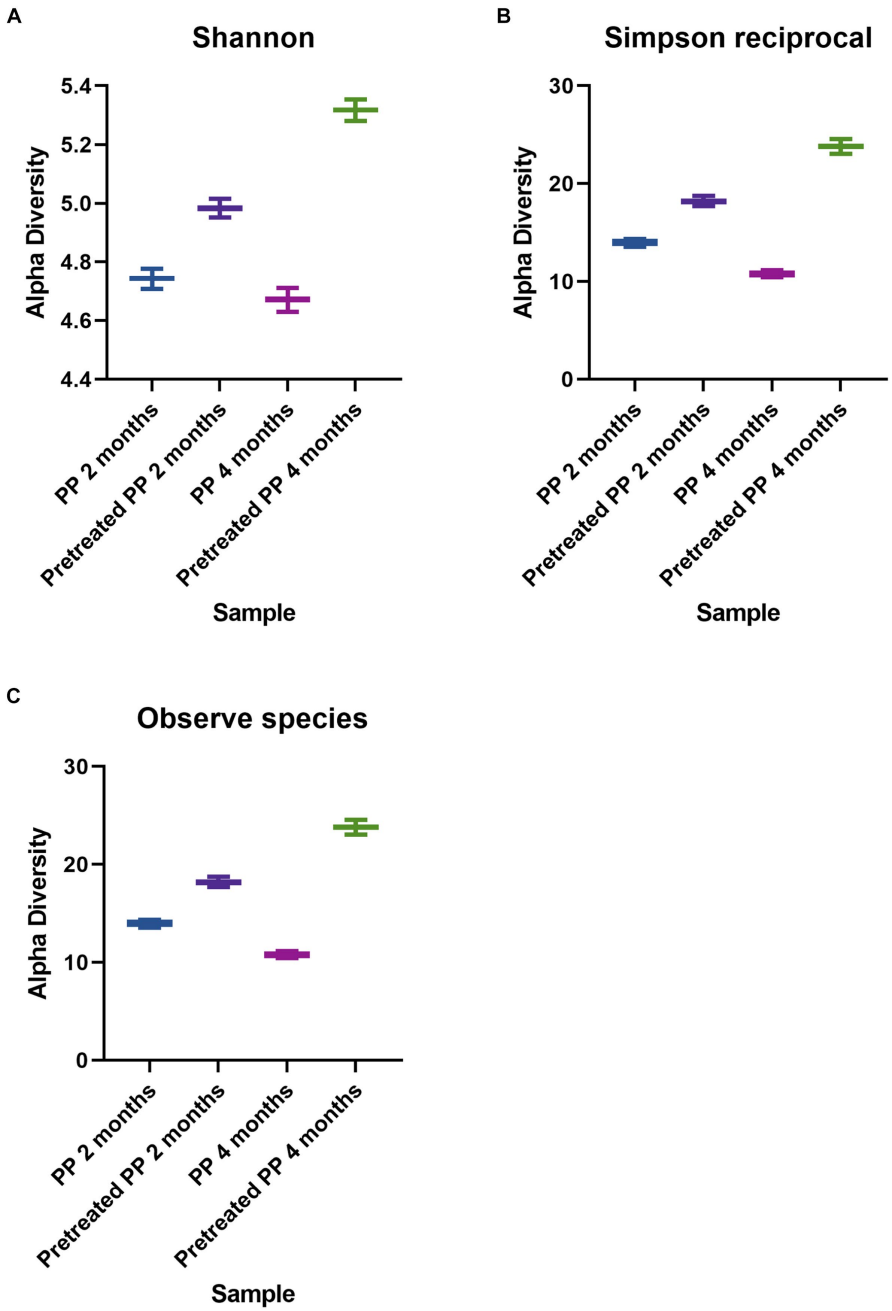
Alpha diversity indices of bacteria. **(A)** Shannon. **(B)** Simpson reciprocal. **(C)** Observed species.

Alpha diversity in each treatment was estimated by the Shannon and Simpson reciprocal statistics (Figures 1A,B), as well as from a direct count of the number of species observed. The indices represent the degree of microbial diversity. Higher Shannon and reciprocal Simpson indices indicate that the microbial community is more diverse. Among the enrichment cultures, the highest bacterial diversity was found in the 4-month culture with pretreated PP. The diversity of the bacterial community of virgin PP at 2 months was higher than at 4 months, while the diversity of pretreated PP enrichment for 2 months was lower than the enrichment culture at 4 months. The results for the numbers of observed species (corresponding to the reciprocal Simpson index) showed that the greatest number of species was found with the pretreated PP enrichment for 4 months, followed by PP enrichment for 2 months, pretreated PP enrichment for 2 months, and PP enrichment for 4 months (Figure 1C).

### 3.2 Bacterial community structure of PP and pretreated PP enrichment culture

**Figure 2:**
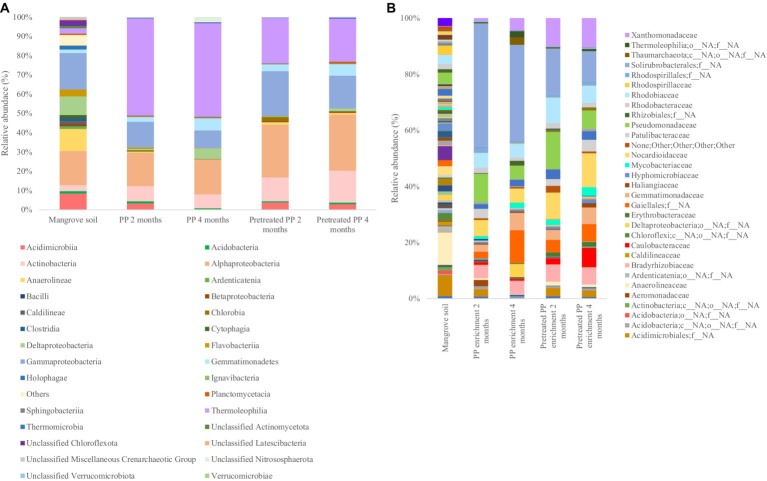
The bacterial community of bacterial biofilm colonizing PP and pretreated PP under enrichment with mangrove soil for 2 and 4 months **(A)** top 30 classes **(B)** top 30 families.

The top 30 bacterial classes in the 2-month and 4-month enrichment cultures are shown in Figure 2A. *Thermoleophilia*, *Gammaproteobacteria*, *Gemmatimonadetes*, *Alphaproteobacteria*, and *Actinobacteria* were the most abundant classes in all samples. In addition, the bacterial community composition at the family level is shown in Figure 2B. The composition of the bacterial community in the 2-month enrichment culture of PP revealed the abundance of one unclassified family within *Solirubrobacterales* (44.2%), *Pseudomonadaceae* (10.2%), *Rhodobiaceae* (5.3%), and *Nocardioidaceae* (5.6%). In the 2-month enrichment culture of pretreated PP, the bacterial community was similar to that of PP but with different proportions. The families that dominated in the 2-month enrichment culture of pretreated PP were one unclassified family within *Solirubrobacterales*, *Pseudomonadaceae*, *Nocardioidaceae*, and *Rhodobiaceae*, accounting for 16.3, 13.2, 9.4, and 9% of the relative abundance, respectively. In addition, *Xanthomonadaceae* was abundant in the 2-month enrichment culture of pretreated PP, accounting for 9.9%.

Moreover, the comparison of bacterial community abundance in pretreated PP with virgin PP in the 2-month enrichment culture revealed an increase in the numbers of certain taxa. Notably, *Xanthomonadaceae* constituted the highest abundance (9.00%), followed by *Rhodobiaceae* (3.80%), *Norcardioidaceae* (3.76%), *Pseudomonadaceae* (3.00%), one unclassified family within *Gaiellales* (2.18%), *Erythrobacteraceae* (1.41%), *Caulobacteraceae* (1.36%), *Bradyrhizobiaceae* (1.33%), *Gemmatimonadaceae* (0.95%), *Anaerolineaceae* (0.83%), *Mycobacteriaceae* (0.75%), one unclassified family within *Acidimicrobiales* (0.37%), *Hyphomicrobiaceae* (0.27%), one unclassified family within *Rhizobiales* (0.17%), *Thermoleophilia* (0.09%), *Rhodobacteraceae* (0.27%), *Gemmatimonadetes* (0.27%), *Xanthobacteraceae* (0.02%), and *Rhodospirillaceae* (0.01%), respectively (Figure 2B).

The initial stage of plastic biodegradation involves the formation of a biofilm. Therefore, bacteria that form biofilms such as *Alphaproteobacteria* and *Gammaproteobacteria* could potentially serve as early colonizers (De Tender et al., 2017). *Rhodobiaceae* belonging to *Alphaproteobacteria* and *Pseudomonadaceae* belonging to *Gammaproteobacteria* were abundant in the 2-month enrichment culture of PP and pretreated PP. In addition, the families *Xanthomonadaceae* and *Bradyrhizobiaceae* belonging to the classes *Gammaproteobacteria* and *Alphaproteobacteria*, respectively, were dominant in the 2-month enrichment culture of pretreated PP. Additionally, genera in the family *Pseudomonadaceae* such as *Pseudomonas* have been reported as potential degraders of various polymers, including PE, PP, PS, and PET (Mohanan et al., 2020; Ru et al., 2020). However, one unclassified family within *Solirubrobacterales* was predominant in both the 2-month enrichment culture of PP and pretreated PP. This family has been previously reported to play a role in carbon metabolism within soil ecosystems (Zhu et al., 2022).

After 4 months of enrichment culture, the abundance of primary biofilm colonizers was decreased. In the 4-month enrichment culture of PP, the dominant families were one unclassified family within *Solirubrobacterales*, one unclassified family within *Gaiellales*, *Gemmatimonadaceae*, and *Nocardioidaceae*, with relative abundances of 34.4, 11.6, 6.1, and 4.9%, respectively. The results indicated that the abundance of one unclassified family within *Gaiellales*, *Gemmatimonadaceae*, *Xanthomonadaceae*, and one unclassified order and family within *Deltaproteobacteria* had increased. Chen et al. (2021) reported that *Gaiellales* are predominant in extreme environments such as mangrove wetlands, saline-alkaline soils, and wastewater treatment plants. However, their specific functions remain unknown due to the lack of indigenous phenotypes. Similarly, the role of *Gemmatimonadaceae* in polymer degradation also remains unclear. In addition, members of *Xanthomonadaceae* such as *Xanthomonas* and *Arenimonas* have been reported for their ability to degrade PET and PS (Ru et al., 2020; Miao et al., 2023). A previous study found that *Deltaproteobacteria* was predominant in the biofilms of floating plastic waste (Delacuvellerie et al., 2019) and the bacterial community involved in PET and biodegradable plastic degradation (Denaro et al., 2020).

The families abundant in the 4-month enrichment culture of pretreated PP were *Nocardioidaceae* (12.1%) followed by one unclassified family within *Solirubrobacterales*, *Xanthomonadaceae*, *Caulobacteraceae*, *Pseudomonadaceae*, and *Rhodobiaceae*, accounting for 11, 10.3, 6.8, 6.5, and 6% of the relative abundance, respectively. Similar to those of the 4-month enrichment culture of PP, the primary colonizers of pretreated PP were decreased after 4 months of enrichment except for the *Nocardioidaceae*. Members of the family *Nocardioidaceae, Nocardioides* have been previously reported for their ability to degrade PHB polymers (Akan et al., 2021) and PE (Amobonye et al., 2021). Furthermore, they have been found in abundance within bacterial communities capable of degrading hydrocarbons (Huang et al., 2021). Additionally, the number of *Nocardioidaceae* (genus *Nocardioides*) increased in pretreated PP compared to virgin PP after the 4-month enrichment culture, accounting for 7.14%. This was followed by *Caulobacteraceae* (6.17%), *Xanthomonadaceae* (6.01%), *Patulibacteraceae* (3.97%), one unclassified family within *Acidimicrobiales* (2.32%), *Pseudomonadaceae* (1.70%), *Erythrobacteraceae* (1.34%), *Mycobacteriaceae* (1.31%), *Rhodobiaceae* (1.22%), *Bradyrhizobiaceae* (1.21%), *Haliangiaceae* (0.99%), *Anaerolineaceae* (0.78%), *Hyphomicrobiaceae* (0.75%), unclassified *Rhodospirillales* (0.55%), *Rhodobacteraceae* (0.52%), *Acidobacteria* (0.34%), *Caldilineaceae* (0.08%), and *Xanthobacteraceae* (0.02%), respectively. Other families that increased in abundance in the 4-month enrichment culture were *Gemmatimonadaceae*, *Xanthomonadaceae*, and *Caulobacteraceae*.

These findings suggest that the community of bacteria capable of degrading plastic was stimulated after the enrichment cultures. Moreover, the dynamic changes in the bacterial community suggest that during the initial stages of biodegradation, the plastic was colonized by biofilm-forming bacteria. Subsequently, the population of bacteria capable of degrading plastic polymers increased and remained persistent within the community.

**Figure 3:**
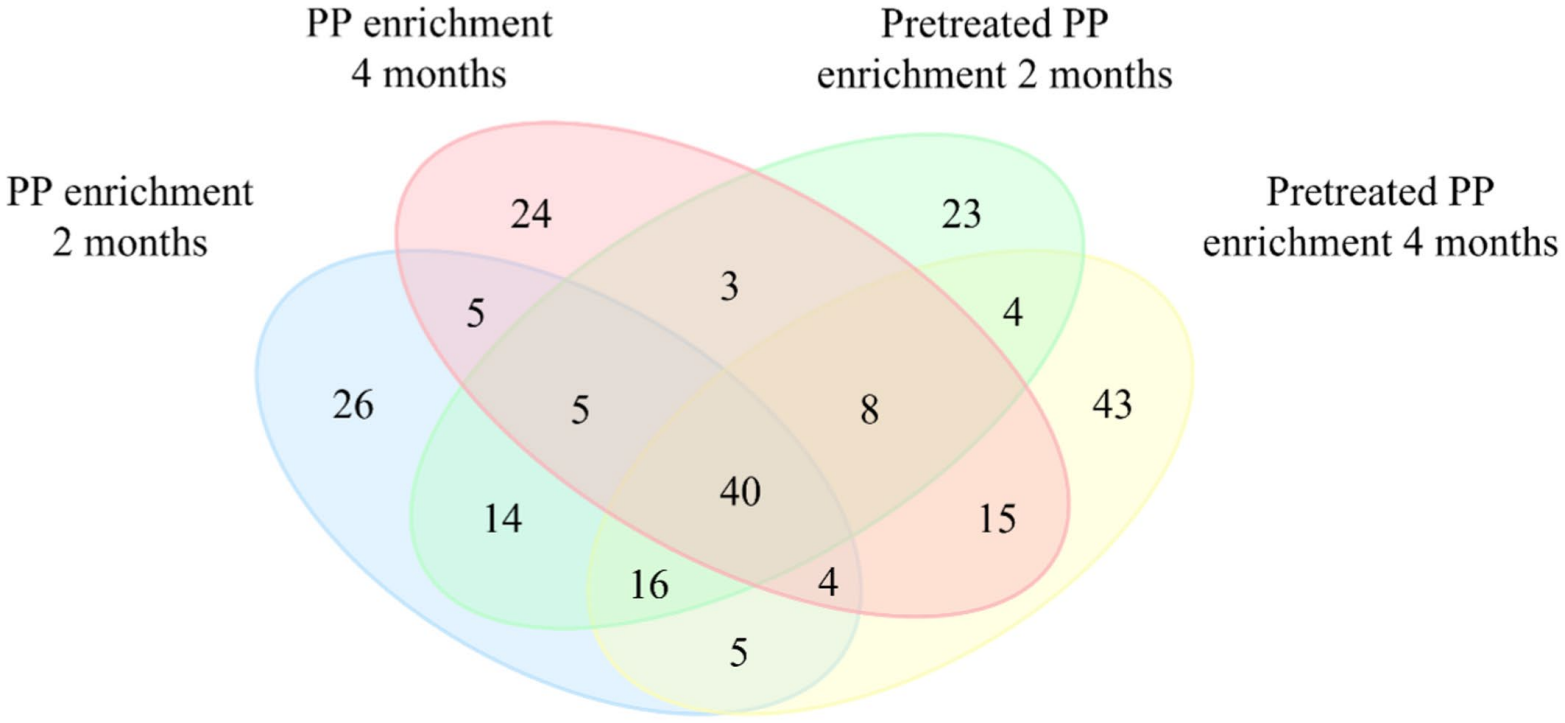
Venn diagram representing the numbers of unique and shared bacterial amplicon sequence variants (ASVs) between four groups of samples. Values within intersections represent a shared sequence, while values outside intersections represent unique sequences.

A Venn diagram was constructed to illustrate the differences in the bacterial community between PP enrichment and pretreated PP enrichment for 2 and 4 months at the amplicon sequence variants (ASVs) level (Figure 3). Forty ASVs were shared among all enrichment samples, while 26, 24, 23, and 43 ASVs were unique in PP and pretreated PP enrichment for 2 months and 4 months, respectively. There were five ASVs shared between the 2-month and 4-month PP enrichment cultures, while there were four ASVs shared between the 2-month and 4-month enrichment cultures of pretreated PP. These findings suggest that these 40 ASVs are bacteria capable of utilizing both PP and pretreated PP or their metabolites as substrates. Future isolation of these bacteria could allow direct testing of their ability to degrade PP. Within this group of ASVs, various genera were represented, including one unclassified family within *Solirubrobacterales*, one unclassified family within *Gaiellales*, *Nocardioides*, *Arenimonas*, *Parvibaculum*, and *Bradyrhizobium*. These bacteria could be potential strains for the biodegradation of PP.

### 3.3 FTIR

The attenuated total reflectance Fourier transform infrared spectroscopy was used to examine the chemical alteration of PP sheets after incubation with mangrove sediment. FTIR measures the absorption or attenuation of electromagnetic radiation caused by the vibration of chemical bonds within molecules (Gopanna et al., 2018). In addition, FTIR can also quantify the number of functional groups in a molecule based on the intensity of the signal (Stuart, 2004). The FTIR spectra for PP are shown in Supplementary Figure S1. To characterize PP, the major transmittance band was found at 808 cm^−1^, which was assigned to the rocking vibration of methylene (−CH_2_). The bands at 841 and 997 cm^−1^ correspond to the rocking vibration of methyl (−CH_3_) groups. The transmittance peaks located at 972 and 1,168 cm^−1^ correspond to C-C chain stretching and −CH_3_ rocking vibration. Symmetric bending vibration of methyl groups (−CH_3_) was found at 1,375 cm^−1^, and symmetric bending of methylene (−CH_2_) was located at 1,455 cm^−1^. In addition, the transmittance bands at 2,838, 2,869, 2,917, and 2,951 cm^−1^ were related to symmetric stretching of methylene (−CH_2_), symmetric stretching of methyl groups (−CH_3_), asymmetric stretching vibration of methylene (−CH_2_), and asymmetric stretching vibration of methyl (−CH_3_), respectively (Nielsen et al., 2002; Gopanna et al., 2018; Negi et al., 2023).

**Figure 4:**
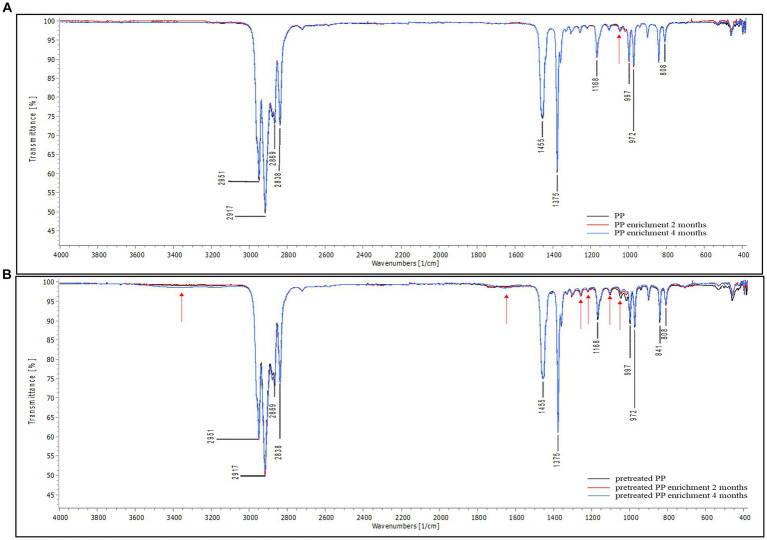
FTIR spectra of PP **(A)** virgin PP and PP enrichment culture for 2 and 4 months **(B)** pretreated PP and pretreated PP enrichment with mangrove soil for 2 and 4 months (black line = control, red line = 2-month enrichment culture, and blue line = 4-month enrichment culture). The red arrow represents the location of a difference in peak intensity in the FTIR spectra.

The results of FTIR analysis are shown in Figure 4 The spectral peaks were normalized to 2,100 cm^−1^ for better differentiation of the changes in spectral intensity. Figure 4A shows the spectra of virgin PP and PP after enrichment for 2 and 4 months. The peak intensity of enrichment samples at the major band is similar to that of the control. However, a slight difference was observed at 1,045 cm^−1^ (red arrow), corresponding to the rocking vibration of the methyl (−CH_3_) group. This suggests that microorganisms induced minimal changes in PP, indicating that the biodegradation of PP is a challenging process due to linear long chains containing methyl (−CH_3_) groups along the molecular chain. This structure poses difficulties for degradation because it lacks polar molecules that microbial enzymes can readily access. In contrast, in the case of pretreated PP, both the control and enrichment samples displayed distinct variation in peak intensities compared to virgin PP (Figure 4B). Chemical pretreatment introduces polar functional groups into the PP polymer chain as shown in Supplementary Figure S2. Differences in peak intensities were observed in the region around 1,600–1,800 cm^−1^ when compared to FTIR spectra of virgin PP and these peaks were assigned to carbonyl groups (Samat et al., 2023). Consequently, in the enrichment cultures of pretreated PP, distinct changes in peak intensity were observed at various positions in comparison to their respective control samples. These differences in peak intensity were found at 1,045, 1,104, 1,220, and 1,256 cm^−1^, in the region spanning 1,600–1,800 cm^−1^, as well as beyond 3,000 cm^−1^. The peak at 1,104 cm^−1^ corresponds to various molecular motions, including C-C chain stretching, −CH_3_ rocking vibration, −CH_2_ wagging, −CH twisting, and -CH bending (Karacan and Benli, 2011). Additionally, the peak at 1,220 cm^−1^ is assigned to the twisting of methylene (−CH_2_), wagging of −CH, and C-C chain stretching (Nielsen et al., 2002). The peak at 1,256 cm^−1^ is associated with the bending of CH and −CH_2_ (Negi et al., 2023). The transmittance region beyond 3,000 cm^−1^ indicates hydroxyl (−OH) groups. These alterations indicate modifications in some functional groups within the polymer chain. The results suggest that microorganisms are more capable of degrading pretreated PP compared to virgin PP.

### 3.4 Water contact angle

**Figure 5:**
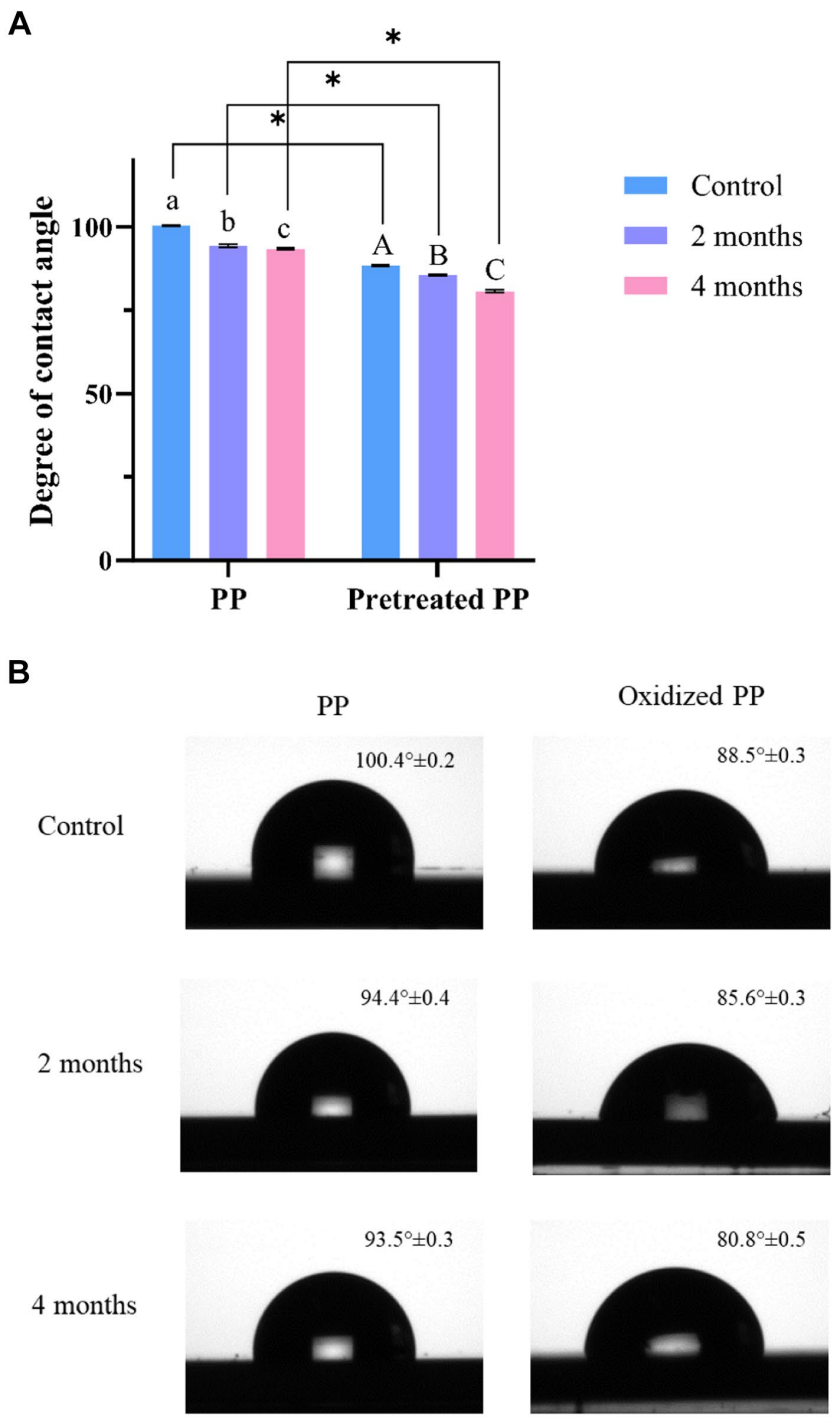
**(A)** Degree of water contact angle measurements of PP and pretreated PP under enrichment with mangrove soil for 2 and 4 months (blue = control, orange = 2-month enrichment culture, and green = 4-month enrichment culture). The error bar represents the result of triplicate measurements. The alphabet represents that the mean difference is significant at the 0.05 level. * represents *p* value < 0.05. **(B)** Image of water contact angle on plastic surface.

The degradation of plastic polymers can also be determined through changes in hydrophobicity. The enzymatic activity of microorganisms either introduces polar functional groups to the polymer chain or damages the surface of the polymer, resulting in a decrease in hydrophobicity. This reduction in hydrophobicity can be measured by the water contact angle. Virgin PP and pretreated PP were used as a control for each group. As shown in Figure 5, the contact angle of virgin PP was 100.4° ± 0.2, indicating that virgin PP surface was highly hydrophobic. The contact angle of pretreated PP was 88.5° ± 0.3, lower than that of PP due to chemical alteration. However, when compared to the control, the contact angles of PP and pretreated PP enrichment culture were significantly decreased (*p* < 0.05) according to the incubation time. The degree of contact angle between water and PP decreased as the incubation time increased, indicating that PP underwent degradation over time.

### 3.5 SEM

**Figure 6:**
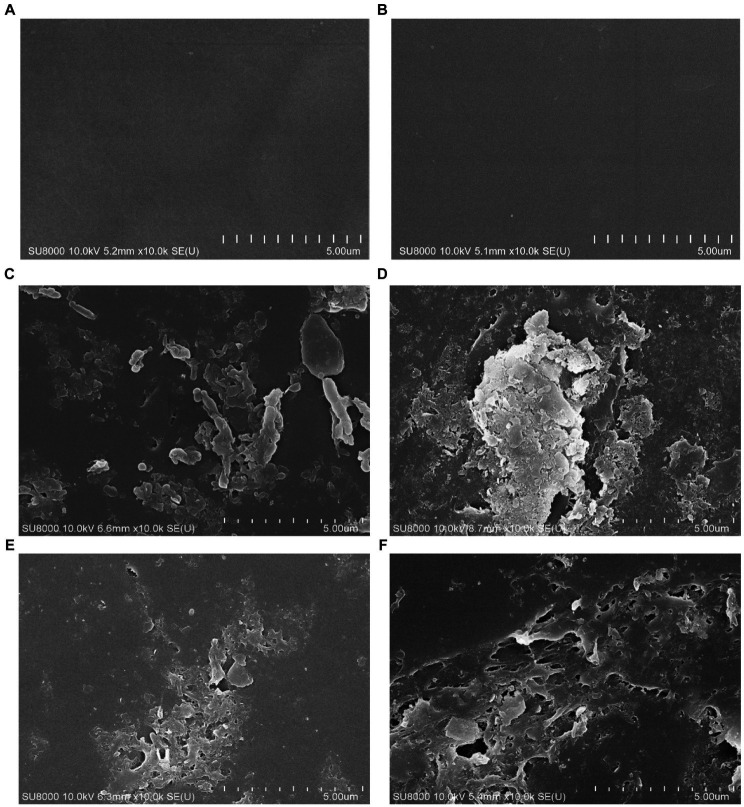
SEM images of PP and pretreated PP enrichment with mangrove soil for 2 and 4 months. **(A)** Virgin PP, **(B)** unincubated pretreated PP, **(C)** 2-month enrichment culture of PP, **(D)** 2-month enrichment culture of pretreated PP, **(E)** 4-month enrichment culture of PP, **(F)** 4-month enrichment culture of pretreated PP.

To confirm the effect of bacterial biodegradation on PP and pretreated PP, the changes in the plastic surface were observed using SEM. After incubation for 2 and 4 months, virgin PP and pretreated PP were recovered from the enrichment cultures, and the surface morphology was observed by scanning electron microscopy (SEM). Figure 6 presents SEM images of different PP and pretreated PP samples. The control samples of virgin PP and pretreated PP showed a smooth surface (Figures 6A,B). In contrast, the SEM images of PP and pretreated PP from the enrichment cultures revealed the presence of bacteria adhering to the plastic surface. In addition, the PP from the enrichment cultures displayed minor cracks and holes. The pretreated PP from the enrichment cultures showed tears, abrasion, cracks, and holes, especially in the 4-month enrichment culture. These findings strongly suggest that bacterial activity contributes to the degradation of plastic.

### 3.6 Plastic-degrading enzymes

**Figure 7:**
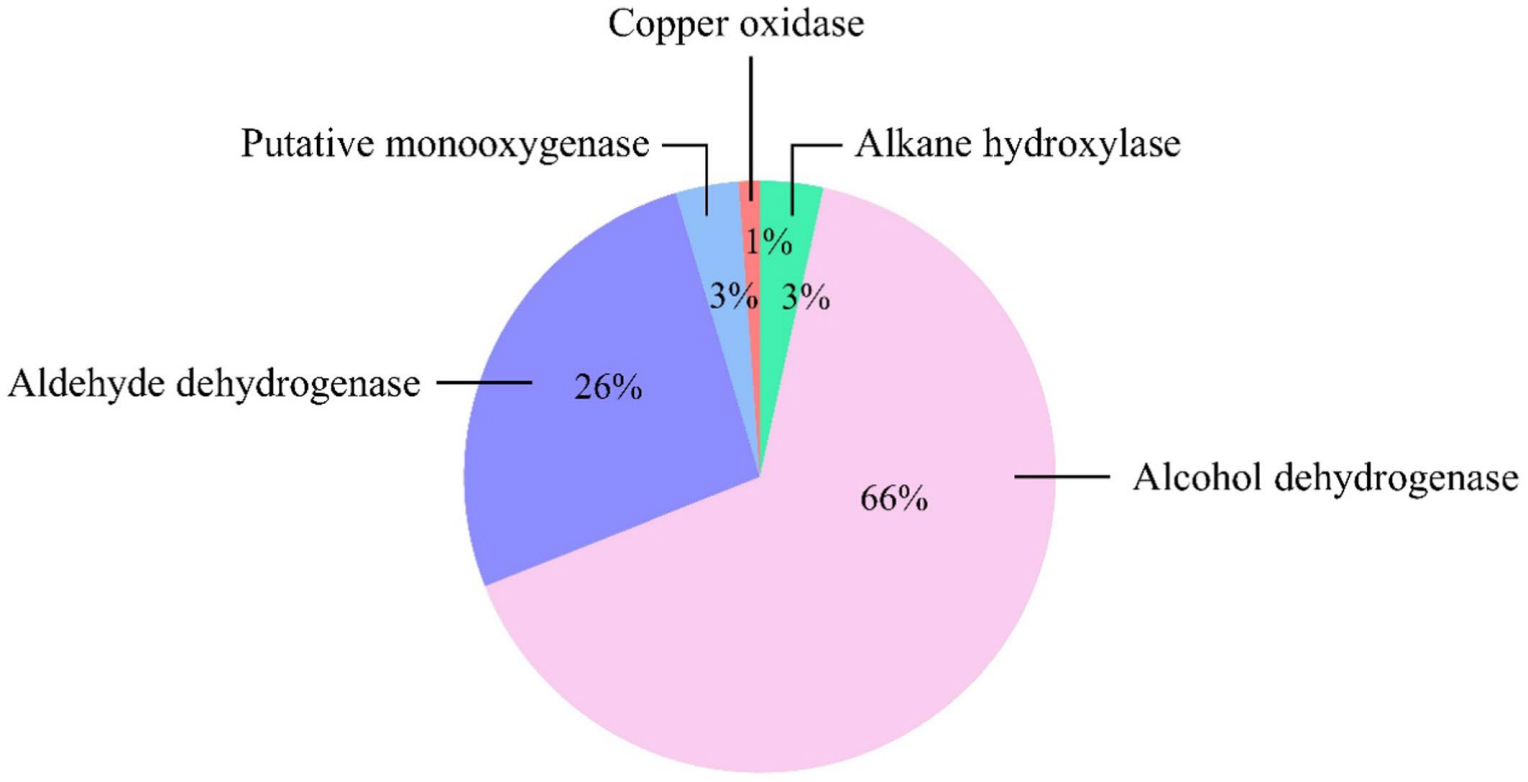
Enzymes potentially degraded C-C backbone polymer in mangrove soil.

Shotgun metagenomics was employed to examine the genes associated with PP-degrading enzymes in mangrove soil. However, no specific enzymes have been reported for PP degradation (Ru et al., 2020). Nevertheless, the structure of PP closely resembles that of PE, with a methyl group (-CH_3_) replacing one of the hydrogens found in the PE structure (Mohanan et al., 2020). Moreover, similar to PE, physiochemical pretreatment such as UV irradiation, thermal oxidation, and blending biodegradable additives could facilitate microbial biodegradation of PP (Ru et al., 2020; Rana et al., 2022). Several plastic-degrading enzymes which have been previously reported in many studies (Mohanan et al., 2020; Ru et al., 2020; Amobonye et al., 2021; Chow et al., 2022) and Pazy database to play a role in the degradation of C-C backbone polymers were observed in Shotgun metagenomics. The results revealed that a total of 87 out of 176,821 contigs were identified as putative C-C backbone polymer-degrading enzymes, including alkane hydroxylase (3 contigs), putative monooxygenase (3 contigs), alcohol dehydrogenase (57 contigs), aldehyde dehydrogenase (23 contigs), and copper oxidase (1 contig) (Figure 7). In addition, these enzymes, including alkane hydroxylase, alcohol dehydrogenase, aldehyde dehydrogenase, and copper oxidase also found in the genome of potential plastic-degrading strains obtained from enrichment culture such as *Nocardioides*, *Arenimonas*, *Parvibaculum*, and *Bradyrhizobium*. To provide insights into the abundance of contig assemblies of the putative plastic-degrading enzymes, the reads were mapped back to the contigs. When consider the top 3 number of enzyme contigs, alcohol dehydrogenase had the highest number of mapped reads. Contig k141_17927, corresponding to alcohol dehydrogenase, exhibited a read count of 11,193, which belonged to the genus *Hartmannibacter* (Supplementary Table S1). Another notable contig, k141_292792 assigned to alkane hydroxylase, was also observed in significant quantities, with a count of 772 reads. The remaining contigs exhibited read counts below 1,000 (Supplementary Figure S3). Among the putative C-C backbone polymer-degrading enzymes, alcohol dehydrogenase was identified as the most abundant enzyme. Pseudomonadota exhibited a high abundance of alcohol dehydrogenase, followed by Actinomycetota and Chloroflexota. According to Ru et al. (2020), this enzyme works in collaboration with alkane hydroxylase and aldehyde dehydrogenase to break down aliphatic hydrocarbons. The process of aliphatic hydrocarbon degradation involves alkane hydroxylase oxidizing the terminal methyl group, converting it into a primary alcohol. Hence, alkane hydroxylase was also detected in the sample, and the genus *Thiohalobacter*, belonging to Pseudomonadota, demonstrated a substantial presence of alkane hydroxylase, as indicated in Supplementary Table S1. In addition, a transcriptomic analysis of *Rhodococcus opacus* R7, growing on a medium supplemented with polyethylene (PE), revealed the up-regulation of the *alkB* gene, which encodes alkane hydroxylase (Zampolli et al., 2021). Moreover, the expression of gene encoding for alkane hydroxylase on bacteriophage can initiate the degradation of long-chain alkane (Ru et al., 2023). In the subsequent step, primary alcohol is then oxidized by alcohol dehydrogenase. Additionally, alcohol dehydrogenase is commonly active in the bacterial fermentation pathway, serving as an energy generation mechanism. This could explain its high abundance in the sample. The products resulting from alcohol dehydrogenase activity are further oxidized by aldehyde dehydrogenase, and finally converted into fatty acids that undergo the β-oxidation pathway and TCA cycle to generate biomass, CO_2_, and H_2_O (Ru et al., 2020). Similar to those of alcohol dehydrogenase, aldehyde dehydrogenase also functions in the fermentation pathway. Additionally, *Methyloceanibacter*, a member of Pseudomonadota, emerged as the primary taxon identified in aldehyde dehydrogenase. This phylum was also prominently associated with alcohol dehydrogenase and alkane hydrolase (Supplementary Table S1).

## 4 Conclusion

Thailand annually produces a significant amount of plastic waste, and the inadequate management of this waste has led to its proliferation in various environments, including marine ecosystems. Microorganisms are capable of decomposing plastic waste in the environment. Thus, the objective of this study was to explore the bacterial community present in mangrove sediment that possesses the ability to degrade PP, along with the associated enzymes. The enrichment cultures of PP and pretreated PP revealed diverse bacterial communities with the ability to degrade polymers. After 2 and 4 months of enrichment, variation in the proportion of plastic-degrading bacteria within the community was observed, suggesting that different bacteria participate in various stages of plastic biodegradation. In addition, the investigation of plastic-degrading enzymes using Shotgun metagenomics revealed the presence of enzymes associated with plastic degradation, including copper oxidase, alkane hydroxylase, alcohol dehydrogenase, and aldehyde dehydrogenase. The evaluation of plastic degradation demonstrated alterations in the plastic polymer, including changes in functional groups and surface morphology. The results of this study suggest that mangroves could be a promising source of microorganisms or enzymes capable of degrading plastic. However, the mechanisms underlying the microbial conversion of PP require further investigation, and the current feasibility of implementing these processes in bioremediation applications remains limited, necessitating additional research in the future.

## Data availability statement

The datasets presented in this study can be found in online repositories. The names of the repository/repositories and accession number(s) can be found in the article/Supplementary material.

## Author contributions

OP: Conceptualization, Data curation, Formal analysis, Investigation, Methodology, Validation, Visualization, Writing – original draft. NJ: Formal analysis, Investigation, Methodology, Validation, Visualization, Writing – review & editing. WS: Conceptualization, Writing – review & editing. PP-G: Data curation, Writing – review & editing. TP: Conceptualization, Writing – review & editing. PP: Conceptualization, Writing – review & editing. PT: Conceptualization, Writing – review & editing. JE: Conceptualization, Supervision, Writing – review & editing. BI: Conceptualization, Supervision, Writing – review & editing.

## Footnotes

^1^ https://pazy.eu/doku.php

^2^ http://www.effemm2.de/spectragryph/
