# Supplementary material for: Exploring untapped bacterial communities and potential polypropylene-degrading enzymes from mangrove sediment through metagenomics analysis
Source: Front Microbiol. 2024 Apr 4;15:1347119. doi: 10.3389/fmicb.2024.1347119 (PMC11024650; doi:10.3389/fmicb.2024.1347119)
Supplement: Supplementary file 1 [file Data_Sheet_1.docx]

**Supplementary data**

**Figure S1:** FTIR spectra of virgin PP with major transmittance peaks.

**s**

**Figure S2:** FTIR spectra of Pretreated PP with major transmittance peaks. The peaks located at 1600-1800 cm^-1^ showed exhibited differences in peak intensities when compared to virgin PP.

**Figure S3:** Count reads of the contigs associated with plastic degrading enzymes re-mapping with De novo assembly (cut off 1,000 reads).

###### Table S1 : PP-degrading enzymes filtering from De novo assembly gene annotated.

| **No.** | **Locus tag** | **Length (bp)** | **Gene** | **EC number** | **Product** | **Target enzymes** | **Organisms** | **Contigs name** | **Mapped reads** |
| --- | --- | --- | --- | --- | --- | --- | --- | --- | --- |
| 152 | JAMPHOOB_00154 | 222 | alkB2_1 | 1.14.15.3 | Alkane 1-monooxygenase 2 | Alkane hydroxylase | *Fulvivirga lutea* strain S481 | k141_39 | 24 |
| 3554 | JAMPHOOB_03598 | 444 | adh_1 | 1.1.1.1 | Alcohol dehydrogenase | Alcohol dehydrogenase | *Anaerolineaceae* bacterium isolate bin1136 | k141_39166 | 26 |
| 4620 | JAMPHOOB_04675 | 666 |  | 1.1.1.1 | Alcohol dehydrogenase | Alcohol dehydrogenase | *Chloroflexi* bacterium isolate bin467 | k141_154677 | 137 |
| 4849 | JAMPHOOB_04904 | 609 |  | 1.1.1.1 | putative zinc-binding alcohol dehydrogenase | Alcohol dehydrogenase | *Methyloceanibacter* sp. wino2 | k141_231366 | 24 |
| 6214 | JAMPHOOB_06285 | 651 | adhT_1 | 1.1.1.1 | Alcohol dehydrogenase | Alcohol dehydrogenase | *Rhodospirillales* bacterium | k141_155054 | 24 |
| 10983 | JAMPHOOB_11108 | 318 | aldh | 1.2.1.3 | Aldehyde dehydrogenase | Aldehyde dehydrogenase | *Deltaproteobacteria* bacterium isolate bin407 | k141_2443 | 18 |
| 11262 | JAMPHOOB_11391 | 375 | acoD_1 | 1.2.1.3 | Acetaldehyde dehydrogenase 2 | Aldehyde dehydrogenase | *Thermoanaerobaculia* bacterium isolate 14c23368-4dca-4d55-9b94-f295180eceef | k141_79051 | 28 |
| 12070 | JAMPHOOB_12208 | 252 | adhA_1 | 1.1.1.1 | putative alcohol dehydrogenase AdhA | Alcohol dehydrogenase | *Rhodospirillales* bacterium isolate bin96 | k141_232773 | 196 |
| 13774 | JAMPHOOB_13930 | 705 | aldA_1 | 1.2.1.3 | Putative aldehyde dehydrogenase AldA | Aldehyde dehydrogenase | *Actinokineospora* sp. UTMC 2448 | k141_3054 | 32 |
| 14716 | JAMPHOOB_14880 | 654 | adhT_2 | 1.1.1.1 | Alcohol dehydrogenase | Alcohol dehydrogenase | *Herbaspirillum* sp. DW155 | k141_118548 | 32 |
| 16831 | JAMPHOOB_17014 | 999 | adhA_2 | 1.1.1.1 | putative alcohol dehydrogenase AdhA | Alcohol dehydrogenase | *Hyphomicrobium* sp. DMF-1 | k141_3703 | 205 |
| 18225 | JAMPHOOB_18417 | 987 | adhA_3 | 1.1.1.1 | putative alcohol dehydrogenase AdhA | Alcohol dehydrogenase | Uncultured bacterium ctg7180000000736 | k141_195938 | 301 |
| 19476 | JAMPHOOB_19682 | 981 | aldHT_1 | 1.2.1.5 | Aldehyde dehydrogenase, thermostable | Aldehyde dehydrogenase | *Anaerolineales* bacterium isolate bin12 | k141_158201 | 150 |
| 20051 | JAMPHOOB_20266 | 549 | adh_2 | 1.1.1.1 | Alcohol dehydrogenase | Alcohol dehydrogenase | No significant similarity found | k141_4344 | 28 |
| 23121 | JAMPHOOB_23365 | 669 | adh_3 | 1.1.1.1 | Alcohol dehydrogenase | Alcohol dehydrogenase | *Iamia* sp. SCSIO 61187 | k141_197054 | 59 |
| 23353 | JAMPHOOB_23602 | 1155 | adhB_1 | 1.1.1.1 | Alcohol dehydrogenase B | Alcohol dehydrogenase | *Iamia* sp. SCSIO 61187 | k141_120513 | 158 |
| 24601 | JAMPHOOB_24864 | 462 | adhT_3 | 1.1.1.1 | Alcohol dehydrogenase | Alcohol dehydrogenase | *Dehalococcoidia* bacterium isolate | k141_274580 | 10 |
| 27143 | JAMPHOOB_27436 | 372 | adhD | 1.1.1.1 | Putative alcohol dehydrogenase D | Alcohol dehydrogenase | *Thalassobaculaceae* bacterium isolate | k141_44074 | 134 |
| 29789 | JAMPHOOB_30107 | 558 |  | 1.1.1.1 | Alcohol dehydrogenase | Alcohol dehydrogenase | *Dehalococcoidia* bacterium isolate | k141_160701 | 19 |
| 32144 | JAMPHOOB_32494 | 534 | adh_4 | 1.1.1.1 | Alcohol dehydrogenase | Alcohol dehydrogenase | *Pseudolabrys taiwanensis* strain CC-BB4 | k141_83428 | 145 |
| 38469 | JAMPHOOB_38887 | 1062 | adh_5 | 1.1.1.1 | Alcohol dehydrogenase | Alcohol dehydrogenase | *Aureimonas mangrovi* strain LMG 31693 | k141_46414 | 326 |
| 41075 | JAMPHOOB_41524 | 441 | alkB2_2 | 1.14.15.3 | Alkane 1-monooxygenase 2 | Alkane hydroxylase | *Flavobacterium* sp. CS20 | k141_278414 | 17 |
| 42637 | JAMPHOOB_43098 | 147 | aldHT_2 | 1.2.1.5 | Aldehyde dehydrogenase, thermostable | Aldehyde dehydrogenase | *Pseudomonas stutzeri* strain NCTC10475 | k141_238857 | 38 |
| 44105 | JAMPHOOB_44578 | 624 |  | 1.1.1.1 | Alcohol dehydrogenase | Alcohol dehydrogenase | *Chloroflexi* bacterium isolate bin26 | k141_9306 | 29 |
| 45941 | JAMPHOOB_46439 | 183 | bphJ | 1.2.1.10 | Acetaldehyde dehydrogenase 4 | Aldehyde dehydrogenase | *Pseudomonas putida* strain BS3701 | k141_239488 | 42 |
| 47508 | JAMPHOOB_48029 | 543 | adhA_4 | 1.1.1.1 | putative alcohol dehydrogenase AdhA | Alcohol dehydrogenase | *Chloroflexi* bacterium isolate bin156 | k141_202120 | 40 |
| 50035 | JAMPHOOB_50583 | 423 | aldHT_3 | 1.2.1.5 | Aldehyde dehydrogenase, thermostable | Aldehyde dehydrogenase | *Anaerolineales* bacterium isolate | k141_280503 | 16 |
| 51877 | JAMPHOOB_52453 | 336 | acoD_2 | 1.2.1.3 | Acetaldehyde dehydrogenase 2 | Aldehyde dehydrogenase | *Methyloceanibacter* sp. wino2 | k141_10964 | 20 |
| 53669 | JAMPHOOB_54260 | 735 | acoD_3 | 1.2.1.3 | Acetaldehyde dehydrogenase 2 | Aldehyde dehydrogenase | *Methyloceanibacter caenitepidi* | k141_241025 | 60 |
| 54022 | JAMPHOOB_54618 | 321 | adhA_5 | 1.1.1.1 | putative alcohol dehydrogenase AdhA | Alcohol dehydrogenase | *Kaistia* sp. isolate MAG 2176 | k141_88163 | 37 |
| 56218 | JAMPHOOB_56837 | 1092 | adh_6 | 1.1.1.1 | putative alcohol dehydrogenase adh | Alcohol dehydrogenase | *Pseudonocardia autotrophica* NBRC 12743 | k141_88632 | 278 |
| 56561 | JAMPHOOB_57187 | 354 | aldHT_4 | 1.2.1.5 | Aldehyde dehydrogenase, thermostable | Aldehyde dehydrogenase | *Anaerolineales* bacterium isolate | k141_204176 | 32 |
| 57137 | JAMPHOOB_57770 | 1002 |  | 1.1.1.1 | Alcohol dehydrogenase | Alcohol dehydrogenase | *Thermoplasmata archaeon* isolate bin738 | k141_88851 | 60 |
| 58748 | JAMPHOOB_59394 | 447 |  | 1.1.1.1 | Alcohol dehydrogenase | Alcohol dehydrogenase | *Methanosarcinales archaeon* ANME-1 ERB7 | k141_282572 | 28 |
| 60246 | JAMPHOOB_60911 | 1026 |  | 1.1.1.1 | Alcohol dehydrogenase | Alcohol dehydrogenase | *Thalassospiraceae* bacterium LMO-JJ14 | k141_282946 | 2691 |
| 61726 | JAMPHOOB_62409 | 354 | adhT_4 | 1.1.1.1 | Alcohol dehydrogenase | Alcohol dehydrogenase | *Mesorhizobium* amorphae CCNWGS0123 | k141_283307 | 34 |
| 64976 | JAMPHOOB_65684 | 927 |  | 1.1.1.1 | Alcohol dehydrogenase | Alcohol dehydrogenase | *Anaerolineaceae* bacterium isolate bin52 | k141_90630 | 44 |
| 69149 | JAMPHOOB_69909 | 972 |  | 1.1.1.1 | Alcohol dehydrogenase | Alcohol dehydrogenase | *Candidatus Aminicenantes* bacterium isolate bin99 | k141_52964 | 55 |
| 70215 | JAMPHOOB_70992 | 687 | adh_7 | 1.2.1.3 | Aldehyde dehydrogenase | Aldehyde dehydrogenase | *Thalassospiraceae* bacterium LMO-JJ14 | k141_91779 | 19 |
| 70257 | JAMPHOOB_71034 | 648 | adhB_2 | 1.1.1.1 | Alcohol dehydrogenase 2 | Alcohol dehydrogenase | *Polymorphum gilvum* SL003B-26A1 | k141_131262 | 30 |
| 70509 | JAMPHOOB_71289 | 162 | adh_8 | 1.2.1.3 | Aldehyde dehydrogenase | Aldehyde dehydrogenase | *Methyloceanibacter caenitepidi* | k141_131319 | 53 |
| 71327 | JAMPHOOB_72119 | 498 |  | 1.1.1.1 | putative zinc-binding alcohol dehydrogenase | Alcohol dehydrogenase | *Bryobacterales* bacterium isolate | k141_170329 | 52 |
| 71646 | JAMPHOOB_72438 | 987 | adhA_7 | 1.1.1.1 | putative alcohol dehydrogenase AdhA | Alcohol dehydrogenase | *Hyphomicrobium* sp. DMF-1 | k141_92114 | 70 |
| 81240 | JAMPHOOB_82136 | 342 | adhA_8 | 1.1.1.1 | putative alcohol dehydrogenase AdhA | Alcohol dehydrogenase | *Anaerolineales* bacterium isolate bin917 | k141_209617 | 59 |
| 81949 | JAMPHOOB_82854 | 1509 | aldA_2 | 1.2.1.3 | Putative aldehyde dehydrogenase AldA | Aldehyde dehydrogenase | *Allorhizobium pseudoryzae* strain DSM 19479 | k141_246885 | 94 |
| 82639 | JAMPHOOB_83553 | 480 | adh_10 | 1.1.1.1 | Alcohol dehydrogenase | Alcohol dehydrogenase | *Alphaproteobacteria* bacterium isolate | k141_94534 | 18 |
| 85870 | JAMPHOOB_86815 | 1038 | adh_11 | 1.1.1.1 | putative alcohol dehydrogenase adh | Alcohol dehydrogenase | *Hartmannibacter diazotrophicus* strain E19T | k141_17927 | 11193 |
| 93248 | JAMPHOOB_94269 | 795 |  | 1.1.1.1 | Alcohol dehydrogenase | Alcohol dehydrogenase | *Acidimicrobiales* bacterium isolate | k141_290776 | 32 |
| 93902 | JAMPHOOB_94933 | 240 | adhA_9 | 1.1.1.1 | Alcohol dehydrogenase 1 | Alcohol dehydrogenase | *Rhizobiales* bacterium NRL2 | k141_175125 | 63 |
| 97913 | JAMPHOOB_98978 | 771 |  | 1.1.1.1 | putative zinc-binding alcohol dehydrogenase | Alcohol dehydrogenase | *Rhodococcus* sp. X156 | k141_137453 | 156 |
| 98865 | JAMPHOOB_99941 | 876 | puuC_1 | 1.2.1.5 | NADP/NAD-dependent aldehyde dehydrogenase PuuC | Aldehyde dehydrogenase | *Hoeflea* sp. IMCC20628 | k141_213446 | 62 |
| 101219 | JAMPHOOB_102311 | 138 | aldHT_5 | 1.2.1.5 | Aldehyde dehydrogenase, thermostable | Aldehyde dehydrogenase | *Mesorhizobium* sp. M4B.F.Ca.ET.058.02.1.1 | k141_176790 | 13 |
| 101727 | JAMPHOOB_102826 | 1113 | alkB1 | 1.14.15.3 | Alkane 1-monooxygenase 1 | Alkane hydroxylase | *Thiohalobacter thiocyanaticus* | k141_292792 | 772 |
| 105339 | JAMPHOOB_106489 | 564 | adh_12 | 1.1.1.1 | Alcohol dehydrogenase | Alcohol dehydrogenase | *Deltaproteobacteria* bacterium isolate bin162 | k141_61094 | 32 |
| 105625 | JAMPHOOB_106782 | 513 | adhA_10 | 1.1.1.1 | putative alcohol dehydrogenase AdhA | Alcohol dehydrogenase | *Caldithrix abyssi* DSM 13497 | k141_214852 | 16 |
| 106984 | JAMPHOOB_108156 | 507 | alkH_1 | 1.2.1.3 | Aldehyde dehydrogenase | Aldehyde dehydrogenase | *Flammeovirgaceae* bacterium isolate H1_BAC1 | k141_100004 | 30 |
| 109361 | JAMPHOOB_110567 | 396 | acoD_4 | 1.2.1.3 | Acetaldehyde dehydrogenase 2 | Aldehyde dehydrogenase | *Methyloceanibacter* sp. wino2 | k141_100523 | 26 |
| 110696 | JAMPHOOB_111911 | 360 | adhB_3 | 1.1.1.1 | Alcohol dehydrogenase 2 | Alcohol dehydrogenase | *Pseudomonas lalucatii* strain R1b52 | k141_215973 | 26 |
| 111975 | JAMPHOOB_113207 | 324 |  | 1.1.1.1 | putative zinc-binding alcohol dehydrogenase | Alcohol dehydrogenase | *Streptomyces venezuelae* strain ATCC 21782 | k141_179154 | 14 |
| 114639 | JAMPHOOB_115902 | 1011 |  | 1.1.1.1 | Alcohol dehydrogenase | Alcohol dehydrogenase | *Peribacillus butanolivorans* strain KJ40 | k141_63149 | 180 |
| 123127 | JAMPHOOB_124473 | 720 | adh_13 | 1.1.1.1 | Alcohol dehydrogenase | Alcohol dehydrogenase | *Kitasatospora aureofaciens* Tu117 | k141_64990 | 44 |
| 125883 | JAMPHOOB_127262 | 567 | adhT_5 | 1.1.1.1 | Alcohol dehydrogenase | Alcohol dehydrogenase | *Acidimicrobiia* bacterium isolate cMAG.1 | k141_182216 | 28 |
| 131297 | JAMPHOOB_132744 | 633 | adh_14 | 1.1.1.1 | Alcohol dehydrogenase | Alcohol dehydrogenase | *Anaerolineales* bacterium isolate bin12 | k141_27249 | 54 |
| 131893 | JAMPHOOB_133344 | 999 |  | 1.1.1.1 | Alcohol dehydrogenase | Alcohol dehydrogenase | *Anaerolineales* bacterium isolate bin917 | k141_220814 | 220 |
| 135439 | JAMPHOOB_136923 | 330 | alkH_2 | 1.2.1.3 | Aldehyde dehydrogenase | Aldehyde dehydrogenase | *Algoriphagus* sp. NBT04N3 | k141_257898 | 32 |
| 136765 | JAMPHOOB_138267 | 288 | aldHT_6 | 1.2.1.5 | Aldehyde dehydrogenase, thermostable | Aldehyde dehydrogenase | *Pukyongiella litopenaei* strain SH-1 | k141_28442 | 110 |
| 138208 | JAMPHOOB_139741 | 471 | adh_15 | 1.1.1.1 | Alcohol dehydrogenase | Alcohol dehydrogenase | *Rhodospirillales* bacterium isolate Fred_18-Q3-R57-64_MAXAC.396_cln | k141_258418 | 92 |
| 138470 | JAMPHOOB_140005 | 165 | puuC_2 | 1.2.1.5 | NADP/NAD-dependent aldehyde dehydrogenase PuuC | Aldehyde dehydrogenase | *Leisingera aquaemixtae* strain M602 | k141_301006 | 12 |
| 140483 | JAMPHOOB_142034 | 567 | adh_16 | 1.1.1.1 | Alcohol dehydrogenase | Alcohol dehydrogenase | *Anaerolineae* bacterium isolate bin1159 | k141_68907 | 38 |
| 142944 | JAMPHOOB_144525 | 1023 | adhA_11 | 1.1.1.1 | putative alcohol dehydrogenase AdhA | Alcohol dehydrogenase | *Methyloceanibacter caenitepidi* strain Gela4 | k141_223228 | 155 |
| 143055 | JAMPHOOB_144637 | 174 | adhA_12 | 1.1.1.1 | putative alcohol dehydrogenase AdhA | Alcohol dehydrogenase | uncultured *Acidimicrobiia* bacterium | k141_223252 | 26 |
| 146043 | JAMPHOOB_147673 | 825 | aldHT_7 | 1.2.1.5 | Aldehyde dehydrogenase, thermostable | Aldehyde dehydrogenase | *Labrys* sp. KNU-23 | k141_259973 | 96 |
| 147323 | JAMPHOOB_148966 | 462 | adh_17 | 1.1.1.1 | Alcohol dehydrogenase | Alcohol dehydrogenase | *Geminicoccaceae* bacterium SCSIO 64248 | k141_224266 | 33 |
| 147369 | JAMPHOOB_149012 | 1110 | adh_18 | 1.1.1.1 | putative alcohol dehydrogenase adh | Alcohol dehydrogenase | *Massilia* sp. REN29 | k141_30740 | 70 |
| 148131 | JAMPHOOB_149781 | 564 | adh_19 | 1.1.1.1 | Alcohol dehydrogenase | Alcohol dehydrogenase | *Nocardioides anomalus* strain HKS04 | k141_30913 | 42 |
| 150234 | JAMPHOOB_151912 | 426 |  | 1.1.1.1 | putative zinc-binding alcohol dehydrogenase | Alcohol dehydrogenase | *Flammeovirgaceae* bacterium 311 | k141_260830 | 41 |
| 157493 | JAMPHOOB_159266 | 360 | adhA_13 | 1.1.1.1 | putative alcohol dehydrogenase AdhA | Alcohol dehydrogenase | *Pseudolabrys* sp. FHR47 | k141_111099 | 30 |
| 157538 | JAMPHOOB_159312 | 927 | adhA_14 | 1.1.1.1 | putative alcohol dehydrogenase AdhA | Alcohol dehydrogenase | *Pseudolabrys taiwanensis* strain CC-BB4 | k141_111109 | 53 |
| 159246 | JAMPHOOB_161041 | 549 | acoD_5 | 1.2.1.3 | Acetaldehyde dehydrogenase 2 | Aldehyde dehydrogenase | *Methyloceanibacter* sp. wino2 | k141_305557 | 28 |
| 159390 | JAMPHOOB_161187 | 423 | aldHT_8 | 1.2.1.5 | Aldehyde dehydrogenase, thermostable | Aldehyde dehydrogenase | *Chloroflexi* bacterium isolate bin26 | k141_73108 | 12 |
| 165188 | JAMPHOOB_167051 | 234 |  | 1.1.1.1 | Alcohol dehydrogenase | Alcohol dehydrogenase | *Cnuibacter physcomitrellae* strain XA(T) | k141_263912 | 24 |
| 165692 | JAMPHOOB_167563 | 984 | adhA_15 | 1.1.1.1 | putative alcohol dehydrogenase AdhA | Alcohol dehydrogenase | *Burkholderia* sp. JSH-S8 chromosome 2 | k141_34792 | 105 |
| 174581 | JAMPHOOB_176556 | 498 | adh_20 | 1.1.1.1 | Alcohol dehydrogenase | Alcohol dehydrogenase | *Anaerolineales* bacterium isolate bin12 | k141_268445 | 18 |
